# Transcatheter Aortic Valve Replacement in Older Adults: Integrating Cardiac Remodeling and Geriatric Syndromes—A Narrative Review

**DOI:** 10.3390/medicina61091515

**Published:** 2025-08-23

**Authors:** Andoni Fernández-González, Rodrigo Molero-de-Ávila, Bernardo Abel Cedeño-Veloz, Elena Fernández-Jarne, Lucia Lozano-Vicario, Raúl Ramallal Martínez, Nicolas Martínez-Velilla, Gonzalo Luis Alonso Salinas

**Affiliations:** 1 Cardiology Department, Navarre University Hospital (HUN), Navarre Health Service (SNS-O). Irunlarrea St 3, 31008 Pamplona, Spain; andoni.fernandez.gonzalez@navarra.es (A.F.-G.); elena.fernandez.jame@navarra.es (E.F.-J.); raul.ramallal.martinez@navarra.es (R.R.M.); 2 Geriatrics Department, Navarre University Hospital (HUN), Navarre Health Service (SNS-O). Irunlarrea St 3, 31008 Pamplona, Spain; rodrigo.molerodeavila.garcia@navarra.es (R.M.-d.-Á.); ba.cedeno.veloz@navarra.es (B.A.C.-V.); lucia.lozano.vicario@navarra.es (L.L.-V.); nicolas.martinez.velilla@navarra.es (N.M.-V.); 3 Navarrabiomed (Miguel Servet Foundation), Navarra Institute for Health Research (IdiSNA), 31008 Pamplona, Spain; 4 Heath Sciences Department, Navarre Public University (UPNA), 31006 Pamplona, Spain; 5 CIBER of Frailty and Healthy Aging (CIBERFES), Instituto de Salud Carlos III, 28029 Madrid, Spain

**Keywords:** transcatheter aortic valve implantation, transcatheter aortic valve replacement, TAVR, aortic stenosis, elderly, frailty, biomarkers, cardiac remodeling, geriatric syndromes

## Abstract

***Background and Objectives:*** Transcatheter Aortic Valve Replacement (TAVR) has revolutionized the management of severe aortic stenosis (AS), offering a less invasive alternative to surgical replacement, which is particularly beneficial for elderly and high-risk populations. This narrative review aims to summarize current evidence regarding TAVR’s clinical outcomes, patient selection, the role of cardiac remodeling, and the impact of geriatric syndromes on procedural success. ***Materials and Methods:*** This review is based on a comprehensive analysis of the peer-reviewed literature indexed in major scientific databases. We included relevant studies addressing TAVR in older adults, focusing on cardiac biomarkers, imaging, patient stratification, and geriatric syndromes, such as frailty, delirium, and sarcopenia. ***Results:*** Evidence indicates that TAVR significantly improves survival and quality of life in elderly patients with severe AS. Advanced cardiac imaging and biomarkers contribute to improved risk stratification and post-procedural management. Geriatric syndromes are prevalent in this population and strongly influence clinical outcomes. Tailored prehabilitation and multidisciplinary approaches are increasingly recognized as critical components of TAVR care. ***Conclusions:*** TAVR is an effective and safe option for older adults with severe AS. Optimal outcomes depend not only on procedural expertise but also on recognizing and addressing the complex interplay between cardiac pathology and geriatric vulnerabilities. A holistic, patient-centered approach is essential to maximize the therapeutic benefits in this growing patient population.

## 1. Introduction

Aortic stenosis (AS) is the most prevalent valvular heart disease in the elderly, and it inexorably progresses from an asymptomatic phase to a severe, life-limiting condition. Its escalating incidence amidst global population aging necessitates highly effective and durable therapeutic strategies [1,2,3,4,5].

Transcatheter aortic valve replacement (TAVR) has fundamentally reshaped the management of severe AS. Evolving from a niche intervention for high-risk patients, TAVR’s consistent safety and efficacy have progressively expanded its indications across the entire surgical risk spectrum, establishing it as a less invasive, yet equally robust, alternative to surgical aortic valve replacement (SAVR) [6,7,8,9,10].

However, in elderly patients, procedural success alone is insufficient for favorable, long-term outcomes. Prognosis is critically modulated not only by AS severity and hemodynamics but also by the patient’s biological status, comorbidity burden, and age-related conditions. Pre-existing maladaptive cardiac remodeling, frailty, sarcopenia, and cognitive vulnerability significantly influence the recovery trajectory, functional capacity, and survival.

A comprehensive understanding of these interacting determinants is paramount for optimizing patient selection, anticipating complications, and tailoring peri- and post-procedural management. This review provides an integrated, contemporary synthesis of TAVR’s role in older adults, encompassing pathophysiological considerations, prognostic implications of cardiac remodeling, utility of circulating biomarkers and advanced imaging, and the clinical impact of geriatric syndromes. Our aim is to underscore the imperative for a multidisciplinary, individualized approach that transcends isolated valve intervention, addressing the complex biological and clinical realities of elderly patients with severe AS.

### Search Strategy

This narrative review was structured into thematic sections, each supported by a distinct evidence search strategy. The first and third sections—discussing current evidence on TAVR and its prognostic implications, as well as its use in the oldest-old population—were based on targeted PubMed searches using appropriate Medical Subject Headings (MeSH), including “Aortic Valve Stenosis” [MeSH], “Transcatheter Aortic Valve Replacement” [MeSH], “Prognosis” [MeSH], “Treatment Outcome” [MeSH], and “Aged, 80 and over” [MeSH], restricted to the last 10 years (search string example: “Aortic Valve Stenosis” [MeSH] AND “Transcatheter Aortic Valve Replacement” [MeSH] AND “Prognosis” [MeSH] AND “Treatment Outcome” [MeSH] AND “Aged, 80 and over” [MeSH]; Filters: last 10 years, Humans, English).

The second section, referring to serum biomarkers, was constructed based on a systematic search in PubMed, Cochrane, Tripdatabase, and Web of Science, including only articles published within the last 10 years and restricted to humans. A different search was performed for each serum biomarker, following the same structure but varying the biomarker name. A PubMed search was performed using MeSH terms (search string example: (“Transcatheter Aortic Valve Replacement” [Mesh] OR “Aortic Valve Stenosis/surgery” [Mesh] OR “Aortic Valve Stenosis/therapy” [Mesh]) AND “pro-brain natriuretic peptide (1–76)” [Supplementary Concept]), repeating the same structure with varying biomarker names. TripDatabase search was performed using the following strategy: (transcatheter aortic valve replacement” OR “tavi” OR “aortic valve stenosis/surgery” OR “aortic valve stenosis/therapy” OR “aortic valve replacement”) AND (“sst2” OR “soluble st2” OR “st2”). Cochrane search was performed with an advanced search with MeSH term descriptor, with the following search strategy: ([Transcatheter Aortic Valve Replacement] explode all trees OR [Aortic Valve Stenosis] explode all trees and with qualifier(s): [surgery—SU]) AND [Growth Differentiation Factor 15] explode all trees. A Web Of Science search was performed with the following structure: (TS = ((“transcatheter aortic valve replacement” OR “tavi” OR “aortic valve stenosis/surgery” OR “aortic valve stenosis/therapy” OR “aortic valve replacement”))) AND TS = (LGALS3 OR GDF-15 OR galectin 3 OR galectin-3). Overall, 22 articles were eligible for the section, and relevant articles that involved biomarker assessment before and after TAVR were preferred. For the cardiac imaging section, a PubMed search was performed, including only articles published within the last 10 years, with the following search string: (“Transcatheter Aortic Valve Replacement” [Mesh] OR “Aortic Valve Stenosis/surgery” [Mesh] OR “Aortic Valve Stenosis/therapy” [Mesh]) AND (“Ventricular Remodeling” [Mesh]) AND “Cardiac Imaging Techniques” [Mesh]). Only articles referring to TAVR or SAVR were eligible. Seven articles were included in the review.

Finally, the section addressing geriatric syndromes and TAVR was developed through a non-systematic review of the literature retrieved from PubMed and Embase, limited to the past 10 years. Article selection prioritized clinical relevance and thematic contributions to the interaction between TAVR and age-related vulnerability. The search included Medical Subject Headings such as “Frailty” [MeSH], “Delirium” [MeSH], “Sarcopenia” [MeSH], “Geriatric Assessment” [MeSH], and “Aged, 80 and over” [MeSH] (search string example: (“Transcatheter Aortic Valve Replacement” [MeSH] AND (“Frailty” [MeSH] OR “Delirium” [MeSH] OR “Sarcopenia” [MeSH] OR “Geriatric Assessment” [MeSH])) Filters: last 10 years, Humans, English).

Although the risk of bias was taken into account when selecting articles, no specific analysis of quality or bias was performed.

## 2. Procedural Outcomes

TAVR has undergone a remarkable transformation, evolving rapidly from rescue therapy to the established standard of care for severe AS in patients across all surgical risk profiles [1,2,3,4,5]. This progression has been driven by significant technological refinements in prosthetic valve design, delivery devices, and procedural imaging guidance, resulting in a marked reduction in procedural morbidity and mortality [5]. Regarding the type of approach used for the intervention, the transapical approach has shown higher all-cause mortality at 30 days and 1 year, with similar medium- and long-term survival rates compared to the transfemoral approach, which is preferred, especially in older populations. Concerning specific complications according to the type of approach, periprocedural acute myocardial infarction was more common with the transapical approach, and vascular complications were more common with the transfemoral approach. There are no significant differences in the incidence of stroke and atrioventricular block [6,7].

Compared with SAVR, extensive large randomized trials and multicenter registries have consistently demonstrated equivalent or even superior outcomes in diverse patient profiles [1,2,8,9]. For instance, 30-day mortality after transfemoral TAVR is now less than 1% in low-risk patients [2]. In the landmark PARTNER 3 trial, which evaluated the balloon-expandable SAPIEN 3 valve, 30-day all-cause mortality was notably lower in the TAVR arm (0.4%) than in the SAVR arm (1.1%) [1]. Similarly, the EVOLUT Low Risk trial, which utilized the self-expanding Corevalve Evolut R/PRO valve, reported a 30-day mortality of 0.5% in the TAVR arm versus 1.3% in the surgery arm [2]. A meta-analysis of seven randomized clinical trials performed in 2022, which included the entire risk spectrum, showed a protective effect in terms of all-cause mortality of TAVR in the first year after implantation versus SAVR [10]. An American real-world, multicenter, descriptive study in low-risk patients by Verkstein et al. (2025) (extracted from the STS/ACC TVT Registry) showed a similar 30-day event rate to those reported in pivotal clinical trials, with a slightly higher 1 year event rate, probably attributable to greater comorbidity [3].

The short- and medium-term outcomes, such as 1-year all-cause mortality, in contemporary low-risk TAVR cohorts range from 1% to 4.6%, consistent across both pivotal trials and real-world analyses [1,2,3]. Furthermore, TAVR is non-inferior to SAVR in low-risk patients at 5 years [4,5]. Specifically, the EVOLUT Low Risk trial showed an overall survival of 86.5% with TAVR compared to 85.1% with SAVR (*p* = 0.47) [2]. The PARTNER 3 trial also reported favorable 5-year mortality rates: 10.0% for TAVR compared to 8.2% for SAVR, with a hazard ratio (HR) of 1.23 (*p* = 0.38), indicating no significant difference between the two approaches [1,5]. In the intermediate surgical risk cohort, similar results were observed in the SURTAVI clinical trial, with a higher mortality rate in both groups but no significant difference between them (*p* = 0.55) [11]. The data from the NOTION clinical trial showed no statistically significant differences in the 10-year all-cause mortality between TAVR and SAVR (*p* = 0.80) [12]. These robust data underscore the durable survival benefits of TAVR and strongly support the expansion of its indications to a broader patient population.

### 2.1. TAVR-Associated Complications

Atrial fibrillation (AF) is a common morbidity, occurring in approximately 7% to 10% of patients undergoing TAVR, with consistent incidence rates across several surgical risk profiles [1,2,8,9]. Notably, the EVOLUT Low Risk trial demonstrated a significantly lower incidence of new-onset AF with TAVR than with SAVR at 30 days (7% vs. 31%) [2]. The PARTNER 3 trial yielded similar findings, reporting 5% for TAVR versus a substantially higher 39.5% for SAVR, highlighting the significant advantage of TAVR in this respect [1]. The NOTION 10-year outcomes reinforce this assertion, showing 74% of new-onset AF in the SAVR group and 52% in the TAVR group (*p* < 0.01) [12].

Stroke following TAVR exhibits a characteristic biphasic distribution, with the majority of events (69%) occurring within the first 48 h, mainly attributed to procedure-related embolisms [13]. Contemporary trials in low-risk cohorts report acute 30-day stroke rates of 0.5–0.6% [1,2]; although these rates are slightly higher in real-world practice, ranging from 2% to 3% [14]. There are no differences in the 30-day stroke rate between transapical and transfemoral accesses [6,7]. The cumulative stroke incidence reaches 4.3% at one year and between 7.8% and 9.5% at five years of follow-up. Reassuringly, the risk of stroke tends to become comparable to that of the general population after 2 years post-procedure [4,14]. Comparing TAVR versus SAVR cohorts in terms of stroke, there was no increased stroke risk after 2 and 5 years in the TAVR cohort (TAVR 6.5% and SAVR 8.5% at 2 years, *p* = 0.05; TAVR 11.9% and SAVR 13.6% at 5 years, *p* = 0.16) [11]. In the long term, there were no significant differences in long-term stroke rates between the TAVR and SAVR groups (9.7% in the TAVR group, 16.4% in the SAVR group, *p* = 0.1), according to the NOTION 10-year outcomes [12].

Permanent pacemaker implantation (PPI) remains the most frequent non-vascular complication associated with TAVR. In low-risk studies, the rates of PPI at 30 days differ based on valve type: 6.6% for balloon-expandable valves (as seen in PARTNER 3) and a higher 17.4% for self-expanding systems (EVOLUT Low Risk) [1,2]. There are no differences in the PPI rate at 30 days between transapical and transfemoral access [6,7]. In the longer term, the 5-year cumulative PPI incidence reaches 27% with self-expanding valves, remarkably higher than 11% after SAVR (HR 2.70; *p* < 0.001) [4]. In the 10-year term, the permanent PPI cumulative incidence of permanent PPI was notoriously higher in TAVR than in SAVR (44.7% versus 14%, *p* < 0.01) [12]. Despite the significant presence of transient LBBB after TAVR (up to 55% according to Leone et al. 2022), its occurrence does not imply higher 1-year all-cause mortality or PPI [15].

### 2.2. Improvement of Symptoms and Functional Recovery

A hallmark of TAVR is the rapid and substantial improvement in the health-related quality of life (HRQoL). Patients typically experience significant increases of over 20 points in the Kansas City Cardiomyopathy Questionnaire (KCCQ) scores at 30 days post-procedure, outpacing SAVR by 13.6 points. While TAVR initially provides a quicker HRQoL improvement, KCCQ scores tend to converge with those after SAVR at 6 months and remain similar at 5 years [4,16]. Functional gains are further demonstrated by improvements in the 6 min walk test, with significant increases after TAVR, particularly among “slow walkers” [17]. Regarding heart failure (HF) symptoms, approximately 70% of patients improved in at least one NYHA functional class category within the first year. Over 75% of patients maintain NYHA I–II status at 5 and 10 years [2,5,12], which reflects sustained functional recovery.

### 2.3. Structural Valve Durability

Ensuring the long-term performance and durability of the transcatheter valve is paramount, especially if the indications of TAVR are extended to younger patients with lower surgical risk. Five-year data consistently demonstrate rates of structural valve degeneration below 3.5% (SVD) and bioprosthetic valve failure below 5%. These rates are comparable to surgical bioprostheses [4,5,11], even after 10 years (moderate to severe structural valve deterioration 15.4% in TAVR and 20.8% in SAVR, *p* = 0.3) [12]. For instance, the PARTNER 3 trial reported transcatheter valve failure rates of 3.3% versus 3.8% for SAVR at five years, reinforcing trust and confidence in TAVR’s long-term durability [5].

Key points:

- TAVR is non-inferior to SAVR in terms of all-cause mortality in the short, medium, and long term.
- Concerning TAVR-associated complications, TAVR has lower atrial fibrillation rates, similar stroke rates, and higher PPI rates than SAVR in the short-, medium-, and long-term.
- TAVR offers rapid and sustained symptomatic improvement in terms of dyspnea and functional recovery, resulting in improved quality of life based on the KCCQ.
- Structural valve durability of TAVR is comparable to that of bioprosthetic valves in the short, medium, and long term.

Clinical implications:

- TAVR is a less invasive and effective treatment for severe AS, improving patient outcomes, extending survival, and enhancing quality of life across diverse risk profiles.

Research Gaps:

- There is still a lack of evidence regarding the selection of patients who can benefit most from TAVR, especially in the elderly population.
- On the other hand, material and technical development and optimization of the patient before and after TAVR will enable even better results to be achieved.

## 3. Cardiac Remodeling

Cardiac remodeling refers to an array of structural and/or functional alterations in the myocardium that occur in response to acute or chronic stress. These changes can ultimately impair cardiac mechanics or even electrical activity, manifesting as either adverse (detrimental) or reverse (beneficial) remodeling changes. Adaptive mechanisms involved in cardiac remodeling have been meticulously described at histological, cellular, and molecular levels, with these mechanisms varying according to the primary insult [18]. Research into these intricate mechanisms is invaluable for several reasons: it aids in diagnosis, facilitates the establishment of therapeutic targets to halt disease progression, and enables objective evaluation of therapeutic responses.

### 3.1. Cardiac Remodeling Assessment Through Serum Biomarkers

The advancement of translational research has been instrumental in elucidating and defining the relationship between cardiac remodeling and circulating biomarkers. Many of these markers, such as N-terminal pro-B-type natriuretic peptide (NT-proBNP) [19] and carcinoembryonic antigen 125 (CA125) [18], are routinely used in the clinical management of HF. Other circulating markers, including soluble suppression of tumorigenicity 2 (sST2), galectin-3, and growth differentiation factor 15 (GDF-15), have also been linked to myocardial remodeling but are not yet standard in routine clinical practice [19]. The utility of these biomarkers for predicting outcomes after TAVR has been well documented [20,21,22,23,24], albeit the evidence in this regard is still weak (mainly based on small unicentric observational studies) and requires further development.

#### 3.1.1. NT-proBNP

NT-proBNP, the pro-peptide of brain natriuretic peptide (BNP), is released by ventricular cardiomyocytes in response to parietal stress from volume and pressure overload [20]. Its active metabolite possesses natriuretic and vasodilatory properties, contributing to the reduction of adverse cardiac remodeling by inhibiting the renin-angiotensin-aldosterone axis (RAS) and sympathetic nervous system (SNS) activity. It plays a pivotal role in the diagnosis and ongoing monitoring of HF [19,24]. In patients undergoing TAVR, both elevated baseline NT-proBNP levels and their subsequent variation after the procedure have significant prognostic relevance [24]. Observational studies by Ribeiro et al. (2014) [25], Koskinas et al. (2015) [26], Seoudy et al. (2019) [27], and Kaneko et al. (2019) [28] indicate that elevated pre-TAVR levels of NT-proBNP and BNP are associated with a worse 2-year prognosis in terms of overall survival and HF admission. This contrasts with the findings of Vale et al. (2018) [29] and Yu et al. (2023) [24], who reported no statistical significance in this regard (these differences can be explained by the fact that most of the studies were small, single-center studies, with variability in comorbidities and in the patient’s condition prior to TAVR; therefore, further research is needed).

Persistently high NT-proBNP levels at discharge are consistently associated with a worse prognosis for all-cause and/or cardiovascular mortality [24,27,28,29]. Moreover, a worsening trend or absence of reduction in NT-proBNP levels after TAVR may imply a poorer prognosis for all-cause and cardiovascular mortality in the medium and long term [24,27,28,29].

In conclusion, systematic monitoring of NT-proBNP levels both before and after TAVR could serve as a valuable tool to assess therapeutic response, although further research is needed in this field, given the limitations of the available evidence.

#### 3.1.2. CA125

CA125 is a glycoprotein released by mesothelial cells of the peritoneum, pericardium, and pleura in response to stress stimuli and acts as a non-specific marker of tissue irritation. Although historically known as an oncological biomarker, it is now widely used in the field of HF to assess decompensation, particularly if it involves pleural effusion or ascites [20]. In patients with AS, elevated levels of CA125 have been correlated with disease severity and adverse outcomes [20,30]. An observational study by Husser et al. (2013) demonstrated that both the baseline elevation of CA125 levels and their longitudinal evolution were statistically significant independent predictors of all-cause mortality and major cardiovascular events (MACE) [30]. Its use in conjunction with other biomarkers, such as Galectin-3, may further enhance the sensitivity and specificity for predicting adverse outcomes [31].

#### 3.1.3. sST2

sST2, a member of the interleukin-1 receptor family, inhibits IL-33/ST2 signaling, thereby preventing hypertrophic adverse remodeling [32,33]. Its involvement in HF is well documented, as elevated concentrations of this biomarker are observed in patients with AS [32]. Several studies have evaluated sST2 as a prognostic tool [34] in patients scheduled to undergo TAVR. Wernly et al. (2017) found an association between elevated baseline sST2 levels and all-cause mortality at 1-year post-TAVR [32]. Stundl et al. (2017) further demonstrated a correlation between sST2 and echocardiographic cardiac remodeling parameters, creatinine, and BNP, as well as its independent association with all-cause mortality in the first year after TAVR. However, it was not found to be superior to the STS score or NT-proBNP as a prognostic tool in their findings [35]. Schmid et al. (2017) described that the addition of sST2 to the STS score in TAVR patients could improve its prognostic value for adverse outcomes [33]. Given that there are still gaps in our knowledge regarding its optimal use, further research on this promising biomarker. For this reason, we recommend caution when using it to predict adverse events following TAVR in association with the STS score.

#### 3.1.4. GDF-15

GDF-15, a cytokine belonging to the transforming growth factor-beta family, exerts effects on inflammation, oxidative stress, and myocardial injury. Experimental studies in mice have shown the anti-hypertrophic effect of this biomarker [21]. Its relevance to both HF and AS has been previously described [34]. Krau et al. (2015) demonstrated, through a prospective study, the association of higher GDF-15 levels with all-cause mortality and MACE in a TAVR patient population, noting an even higher prognostic value than NT-proBNP [36]. These results were further supported by Kim et al. (2017) [37], who reported that this biomarker offered greater prognostic value than baseline echocardiographic parameters (including left ventricular end-diastolic diameter and volume, left ventricular septal and free wall thickness, heart mass index, left ventricular ejection fraction, left ventricular longitudinal strain, E’ and A’ waves, and E/E’ ratio) when combined with surgical risk prediction scales such as the STS score. They also noted that higher baseline GDF-15 values correlated with less favorable reverse cardiac remodeling post-TAVR [37]. A more recent work by Sluka et al. (2023) suggested that combining GDF-15 and the STS score does not significantly increase prognostic value beyond that provided by baseline GDF-15 alone [38]. Further research is needed to assess the optimal utility of this biomarker in predicting adverse outcomes.

#### 3.1.5. Galectin-3

Galectin-3 is a lectin involved in the signaling pathways related to cell adhesion, cell proliferation, and inflammation. It has been described as a marker of myocardial fibrosis, hypertrophy, and inflammation, and is associated with worsening and adverse events in chronic HF. Regarding AS, clear evidence of its standalone prognostic value remains somewhat debated. According to Baldenhofer et al. (2014), elevated levels of this biomarker prior to TAVR are independently related to a higher rate of adverse outcomes and can enhance prognostic value when associated with NT-proBNP [22]. However, another study by Lindman et al. (2015) did not find an association between higher Galectin-3 levels and cardiovascular adverse events [39,40]. Finally, Rheude et al. (2019) describe that Galectin-3 can predict adverse events in TAVR patients only in the presence of elevated NT-proBNP values [31]. In summary, although this biomarker appears to be useful when used in conjunction with NT-proBNP to assess adverse events after TAVR, we recommend caution in its use, given that additional research is needed in this area [22,39,40,41].

#### 3.1.6. Utility of Serum Biomarkers

The usefulness of the markers described and the best strategy for introducing them into clinical practice remain unclear. The evidence cited highlights the isolated use of NT-proBNP levels after TAVR and the NT-proBNP response comparing pre- and post-TAVR levels for the prediction of adverse events, while the usefulness of pre-TAVR values in isolation for stratifying patients according to prognosis remains unclear [24,25,26,27,28,29]. With regard to CA125, its usefulness in stratifying patients according to prognosis prior to TAVR and the evolution of this biomarker after the procedure to predict adverse events is emphasized, especially when associated with Galectin-3 [30,31]. Concerning sST2, it seems that the best strategy for its use is for follow-up during the first year after TAVR, although its baseline values associated with the STS score prior to the procedure may have prognostic implications [32,33,34,35]. GDF-15 is a promising biomarker, both in combination with surgical risk scales and on its own prior to TAVR, and in isolation after the procedure for predicting adverse events, although further research is needed [36,37,38]. Finally, galectin-3 can be used to predict outcomes prior to TAVR, as long as baseline NT-proBNP values are elevated [39,40,41]. Table 1 summarizes the origin, function, and prognostic value of each biomarker in patients undergoing TAVR.

The proposal to utilize panels of multiple biomarkers has gained traction, as described by Oury et al. (2018) [34] and Lindman et al. (2015). In a 2015 publication by Lindman et al., eight biomarkers were studied (GDF-15, sST2, NT-proBNP, Galectin-3, ultra-sensitive troponin I, myeloperoxidase, ultra-sensitive C-reactive protein, and chemotactic monocyte protein-1); the combination of GDF-15, sST2, and NT-proBNP emerged as the best predictor of all-cause mortality at 1 year post-TAVR [39]. Although further research is needed in this area, the use of multiple serological biomarker panels is recommended for the study of cardiac remodeling, as well as their association with cardiac imaging markers and surgical risk scores, as they have prognostic implications in terms of all-cause mortality [33,35,37,39,41].

### 3.2. Cardiac Remodeling Assessment Through Cardiac Imaging

Beyond biochemical markers, cardiac imaging plays a crucial role in assessing cardiac remodeling in patients undergoing TAVR (Table 2). Post-TAVR improvements have been consistently demonstrated in parameters such as left ventricular end-diastolic or end-systolic diameters and volumes, left ventricular mass index, and various parameters of diastolic function, including the mitral E wave and its relation to the medial and/or lateral E’ wave [40].

Several meta-analyses have emphasized the significant prognostic value of baseline left ventricular global longitudinal strain (LVGLS), assessed by either computed tomography (CT) or transthoracic echocardiography, in predicting all-cause mortality and MACE prior to TAVR [41,42,43,44]. A meta-analysis by Xiangui et al. (2024) further described the utility of baseline extracellular volume fraction (ECV) as a predictor of these events [42], while Xiao et al. (2022) proposed the use of right ventricular longitudinal strain (RVLS) as a prognostic tool for similar outcomes [43]. The variation in LVGLS before and after TAVR has also been identified as an independent prognostic tool for all-cause mortality, as reflected in the meta-analysis by Stents et al. (2022) [44]. An observational study by Anastasius et al. (2022), included in the latter meta-analysis, further demonstrated the prognostic value of changes in left atrial (LA) strain, independent of atrial diameter or volume [45].

As we have presented different minimally invasive tools, it seems reasonable to use serological biomarkers [33,35,37,39] and individualized imaging techniques [41,42,43,44,45] together for a multimodal assessment of cardiac remodeling, although it is necessary to continue with this promising line of research in order to choose the optimal strategy for prognosis stratification prior to TAVR and subsequent prediction of adverse events.

In the near future, artificial intelligence (AI) is expected to play a pivotal role in establishing prognostic clusters and comprehensively assessing cardiac remodeling response to treatment. This will likely be achieved through sophisticated mathematical modeling and machine learning algorithms, as indicated by the recent work of Asheghan et al. (2023) and Meredith et al. (2025) [46,47]. However, it is important to note that further research in this exciting area is still needed.

Key points:

- Baseline levels and post-TAVR evolution of serum biomarkers of cardiac remodelling, such as NT-proBNP, CA125, GDF-15, sST2, and Galectin-3 may play a key role due to their prognostic implications in terms of survival.
- Serum biomarkers can be combined with each other and with surgical risk scales, such as the STS score, to obtain better prognostic stratification of patients.
- Cardiac remodelling parameters obtained through cardiac imaging, such as LVEDD, LVESD, LVEDV, LVESV, LVMI, maximum mitral E wave velocity, medial or lateral e’ wave, E/e’ ratio, LVGLS, RVLS, LA deformation, and ECV, have been shown to have prognostic implications post-TAVR in terms of all-cause mortality and MACE.

Clinical implications:

- Assessment of cardiac remodeling through serum biomarkers should be performed, as it is a non-invasive approach with prognostic implications.
- The combination of serum biomarkers (GDF-15, sST2, and NT-proBNP) and surgical risk scales (sST2 associated with the STS score) improves the ability to predict adverse events.
- The concomitant use of serological markers and cardiac imaging parameters together is reasonable for the assessment of cardiac remodeling.

Research Gaps:

- Although this is a promising field, further evidence is needed to support the use of serological biomarkers for cardiac remodelling assessment.
- Additional high-level evidence studies are needed to select the optimal assessment strategy for cardiac remodelling by combining serological markers and imaging techniques.

## 4. TAVR in Elderly Patients

It is widely recognized that the prevalence of AS significantly increases with age, mainly because the most common cause in developed countries is the valve degenerative process. Indeed, AS has now become the most common heart valve disease in developed countries. The estimated prevalence in patients over 80 years old is between 12.4% and 22.8%, according to several reports [48,49,50]. The overall prevalence of AS may continue to rise in the upcoming years due to the progressive aging of the population and increasing life expectancy.

Since its ideation and development, TAVR has been inherently linked to patients with high or prohibitive surgical risks. This profile of patients is largely associated with advanced age, consistently highlighted in pivotal trials that reported a mean patient age of 82–84 years, with a standard deviation of 7–9 years. Despite this initial conception, there is now robust and comprehensive evidence of effectiveness in patients across a broader spectrum of surgical risks. Consequently, the rate of TAVR has experienced continuous growth, reaching approximately 78,000 procedures annually in the US for symptomatic AS [51].

### 4.1. Procedural Outcomes in Elderly Patients

While comprehensive randomized clinical trials specifically focusing on the “very elderly” patient group (e.g., nonagenarians) are still limited, their inclusion in various observational studies and registries has steadily increased. It provides insights into the safety and effectiveness of TAVR in this specific profile of patients. As early as 2015, a remarkable North American study compared 136 patients aged over 90 years (mean age 92.4 years) with 598 patients under 90 years from the same cohort (mean age 79.7 years). The research showed similar outcomes in terms of procedural success, 30-day and one-year mortality, and major complication incidence. There was only a higher rate of minor vascular complications in the nonagenarian group [52].

Further supporting these findings, a more recent German series included 2336 patients recruited between 2013 and 2018 and compared the outcomes of 153 patients aged over 90 years (6.6% of the total cohort) with the rest of the population sample. It revealed similar rates of key periprocedural complications, such as stroke, the need for PPI, acute kidney injury (AKI), anemia, major bleeding, and residual aortic insufficiency [53].

However, several studies have shown opposite results. A meta-analysis between 2012 and 2017, which encompassed five studies (including 25,371 patients over 90 years compared to 21,442 younger patients), demonstrated significantly poorer outcomes in the nonagenarian group with higher 30-day and 1-year mortality rates. It also showed higher rates of vascular complications, major bleeding, and stroke in the very elderly population, with no differences in the rates of PPI or AKI. Despite these findings, the authors of this meta-analysis concluded that the observed TAVR outcomes were still acceptable and superior to conservative treatment in the nonagenarian cohort, considering the significantly higher surgical risk, life expectancy, and overall quality of life [54].

A more recent meta-analysis provided a comprehensive overview of 23 studies between 2012 and 2019, involving 78,858 patients, of whom 16,094 were nonagenarians (20.4%). It demonstrated notably lower perioperative mortality (6.1%) in the nonagenarian subgroup than the 10% calculated by the STS score. Furthermore, the 1-year mortality rate after TAVR was 20.5%, similar to that of the general population. This sharply contrasts with the estimated 40% mortality rate of conservative management in this cohort. Consequently, these findings strongly suggest that in carefully selected patients, TAVR may be a viable and beneficial option, even in advanced age [55]. Table 3 provides a summary of key comparative studies evaluating procedural outcomes of TAVR in nonagenarians.

### 4.2. Geriatric Syndromes and TAVR

Geriatric syndromes are a significant consideration in the context of TAVR, affecting both patient selection and post-procedural outcomes. Frailty, delirium, and sarcopenia are particularly relevant.

#### 4.2.1. Frailty

Frailty is a reduction in functional reserve and resistance, consequently increasing vulnerability to a range of adverse health outcomes. Several methods and scales have been developed for screening and diagnosis, such as the Fried Frailty Phenotype [56] and the Clinical Frailty Scale (CFS) by Rockwood [57], which are among the most widely recognized scales. Additionally, specific tools such as the Essential Frailty Toolset (EFT) [58] have been developed and validated for use in TAVR patients.

Two published works, including systematic reviews with meta-analyses, have thoroughly examined the association between frailty and outcomes in older adults undergoing TAVR. Thongprayoon et al. (2016) [59] included eight studies with 10,948 patients, and revealed that preoperative frailty doubled the risk of mortality within 1-year post-TAVR (HR = 2.01; 95%CI: 1.44–2.80). However, only two of the included studies used validated frailty scales, and frailty was solely defined by slow gait speed in the most influential study, which included 8039 patients. The second meta-analysis, conducted by Huang et al. (2018) [60], included 14 studies with 7489 participants and identified an increased 6-month mortality rate in frail individuals (HR 2.81; 95%CI: 1.90–4.15). Subgroup analyses demonstrated consistent results when the Fried phenotype was used (HR, 2.69; 95%CI: 2.06–3.50). Thus, frailty was significantly associated with increased 30-day mortality (HR = 2.03; 95%CI: 1.63–2.54), AKI (HR = 1.41; 95%CI: 1.02–1.94), and major bleeding (HR = 1.48; 95%CI: 1.20–1.82). Importantly, this analysis included studies that used more consistent definitions of frailty, mainly employing either the Fried phenotype or the CFS, although one study defined frailty based on a Katz Index score <6 [61].

Regarding the most suitable frailty assessment method, an observational study by Afilalo et al. (2012–2016) [58] demonstrated that frail patients consistently experienced worse 1-year outcomes, encompassing both mortality and disability, regardless of the specific instrument used for assessment (whether the Short Physical Performance Battery [SPPB] [62], Fried phenotype, CFS, or EFT). Among these tools, the EFT showed the strongest association with overall adverse outcomes (OR = 3.72; 95%CI: 2.54–5.45), while 30-day mortality was most strongly predicted by SPPB (OR = 4.07; 95%CI: 1.41–2.71), followed by EFT.

#### 4.2.2. Delirium

Delirium is an acute syndrome characterized by disturbances in attention and cognition. It is particularly relevant in older patients undergoing interventions such as TAVR, as it is associated with a spectrum of worse clinical outcomes, including increased morbidity and mortality and diminished postoperative quality of life.

The incidence of delirium after TAVR was comprehensively analyzed in a meta-analysis by Ochani et al. (2023) [63], which included 42 studies with 47,379 patients. This review reported an overall delirium incidence of 10.5% (95%CI: 9.2–11.9%). A subgroup analysis, stratified by access route, revealed significantly higher delirium rates in patients undergoing non-transfemoral access (transapical, subclavian, direct aortic, or transcaval) at 25.3% (95%CI: 15.4–35.1%) compared to those with transfemoral access at 9.3% (95%CI: 7.6–11.0%). This notable difference may stem from variations in patient profiles, comorbidities, or factors inherent to the more invasive nature of non-transfemoral approaches, such as increased pain or the iatrogenic effects of necessary painkillers.

Another extensive meta-analysis by Ma et al. (2023) [64], which encompassed 70 articles and 413,389 patients, found a mean incidence of 9.8% (95%CI: 8.7–11.0%). However, this rate increased to 20.7% (95%CI: 17.8–23.7%) in studies that employed validated assessment tools with daily screening over the 2-day period after TAVR. Identified risk factors for delirium included non-transfemoral access, general anesthesia, prolonged mechanical ventilation, and AKI. The impact on mortality was examined across 15 studies, which consistently showed that delirium was associated with increased mortality (HR = 2.20; 95%CI: 1.79–2.71), both within the first year and in the longer term.

#### 4.2.3. Sarcopenia

Sarcopenia is closely linked to aging and a sedentary lifestyle. Sarcopenia is defined as the progressive loss of both the quantity and quality of muscle mass and strength. This condition is frequently associated with frailty [65]. A meta-analysis by Soud et al. (2019) [66], which included eight studies with 1881 patients, utilized objective metrics such as psoas muscle area and skeletal muscle mass index (SMI) to assess sarcopenia. The findings indicated that patients without sarcopenia experienced significantly lower long-term mortality (OR 0.49 [95%CI 0.28–0.83]). Moreover, a trend of lower mortality was found in the short term, although significance was not achieved.

A recent study by Solla-Suarez et al. (2024) [67] within the FRAILTY-TAVR cohort further explored the related condition of osteo-sarcopenia (defined as sarcopenia and concomitant osteoporosis) using pre-procedural CT scans of 605 patients. Among them, 91 patients were diagnosed with osteo-sarcopenia, and 126 had a low psoas muscle area. The presence of osteo-sarcopenia was significantly associated with higher 1-year mortality (OR, 3.18; 95%CI: 1.54–6.57) and a greater extent of functional decline (OR, 2.11; 95%CI: 1.19–3.74).

**Figure 1:**
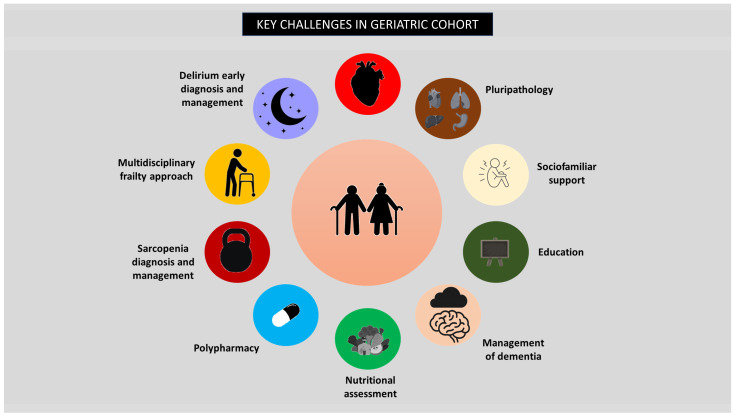
Key challenges in the geriatric cohort of patients undergoing TAVR.

An observational study by Damluji et al. [68] specifically assessed quality of life using the KCCQ in 300 TAVR patients stratified by SMI quartiles. Patients in the lowest SMI quartile (Q1) exhibited significantly worse post-TAVR KCCQ scores than those in the highest quartile (Q4), with a mean difference of 18 points (*p* = 0.02). This difference increased to 23 points (*p* = 0.019) even after adjusting for body mass index (BMI) and cardiovascular risk factors, underscoring the independent impact of sarcopenia on quality of life. Figure 1 summarizes the key challenges faced by geriatric patients undergoing TAVR.

### 4.3. Socioeconomic Impact of Geriatric Syndromes

The economic burden associated with geriatric syndromes in the post-TAVR setting is significant. A retrospective study conducted by Kwak MJ et al. (2020) [69] showed that both delirium and frailty significantly increased the length of hospital stay and associated costs. Interestingly, dementia did not appear to independently increase either of these metrics. The most substantial cost increase, almost 50%, was observed in patients afflicted with both delirium and frailty. Within this context, delirium is postulated to be the most significant independent driver of increased costs.

### 4.4. Quality of Life

In the geriatric patient population, perceived health and patient-centered priorities are paramount outcomes. Thus, we reviewed the relevant literature concerning quality of life in TAVR patients.

An integrative review of 12 studies by Moreines et al. (2024) [70] addressed various concerns expressed by elderly patients undergoing TAVR. This review highlighted that individualized care plans were associated with a perceived better quality of life, even in cases where objective functional improvement was limited. Crucially, patient involvement in decision-making and access to clear and realistic information tailored to their functional status were identified as key factors. While significant improvement in physical symptoms was noted, typically within the first month post-intervention, psychological improvement was also commonly reported. When patients did not experience improvement, they often attributed this to symptoms related to other chronic conditions, a finding that may be partially explained by variations in rehabilitation procedures.

The 5-year follow-up of the CoreValve US Pivotal Extreme Risk Trial (Arnold et al., 2021) [71] provided important long-term quality of life data. Although the trial showed a combined mortality or severe stroke rate of 72.6%, HRQoL (assessed by the KCCQ score) demonstrated substantial improvement. KCCQ scores increased by 24.8 points (95%CI: 22.4–27.2) at 1 year and remained 14.3 points above baseline at five years, despite a gradual decline over time, demonstrating a sustained benefit.

Key points:

- Although the incidence of all-cause mortality and periprocedural complications may be higher in the very elderly, the prognostic and symptomatic benefits are superior to conservative treatment in well-selected patients.
- Geriatric syndromes like frailty, delirium, and sarcopenia are independent determinants of post-procedural outcomes, increasing morbidity and mortality in the short, medium, and long terms.
- Frailty and especially delirium significantly increase hospital length of stay and associated costs.
- TAVR in elderly patients clearly implies rapid and sustained benefits for HRQoL.

Clinical implications:

- In the elderly population, TAVR may be beneficial for carefully selected patients.
- Geriatric syndromes should be prevented, detected early, and addressed, as they lead to worse outcomes and higher overall costs.

Research Gaps:

- Evidence regarding procedural outcomes in very elderly patients (aged ≥90 years) is limited. More high-quality studies are needed to evaluate endpoints, such as all-cause mortality and periprocedural complications, in this population.
- Further research is needed to optimize non-invasive management in elderly patients deemed unsuitable for TAVR, with the goal of improving all-cause mortality, functional status, and quality of life.

## 5. Future Perspectives

The evolving landscape of TAVR continues to drive research to optimize outcomes, particularly for vulnerable geriatric populations. A key area of current investigation focuses on post-procedural interventions designed to mitigate the impact of geriatric syndromes. One notable ongoing trial is PERFORM-TAVI (Protein and Exercise to Reverse Frailty in Older Men and Women Undergoing TAVI) [72]. This study is actively investigating whether a structured program of post-procedural physical training combined with nutritional supplementation can significantly improve clinical outcomes in older frail patients who have undergone TAVR. Addressing frailty through targeted interventions is crucial for enhancing the overall well-being and recovery of this patient cohort.

Complementing this, the Spanish TELE-FRAIL TAVI study [73] is designed to analyze the effect of a telematics-based intervention on frailty reversal in patients over 75 years old with severe AS treated with TAVR. This study, which randomizes patients before hospital discharge, aims to assess whether nutritional support, supervised physical exercise, and health education delivered remotely can improve functional status.

**Figure 2:**
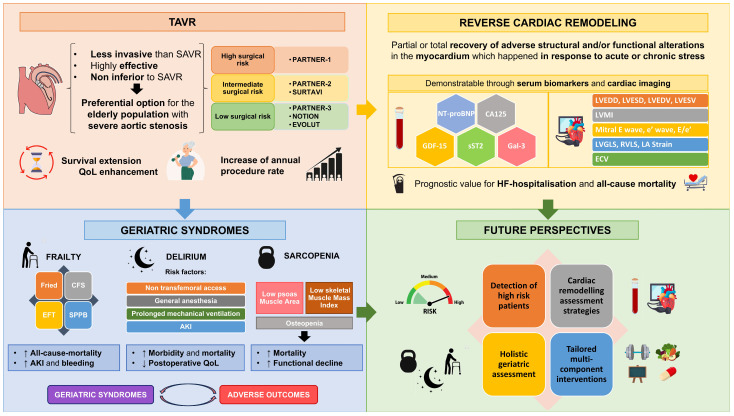
Insights and global overview of TAVR in older adults. TAVI: Trans-aortic valve implantation. SAVR: Surgical aortic valve replacement. QoL: Quality of life. NTproBNP: Pro-peptide of brain natriuretic peptide. BNP: Brain natriuretic peptide; CA 125: Cancer antigen 125. GDF-15: Growth differential factor-15. SST2: Soluble isoform of Suppressor of Tumorigenicity 2. LVEDD: Left ventricular end-diastolic diameter. LVESD: Left ventricular end-systolic diameter. LVEDV: Left ventricular end diastolic volume. LVESD: Left ventricular end systolic volume. HF: Heart failure. LV: Left ventricle. LVMI: Left ventricle mass index. LVGLS: Left ventricular global longitudinal strain. ECV: Extracellular volume. LA: Left auricle. RVLS: Right ventricle longitudinal strain. CFS: Clinical Frailty Scale. EFT: Essential Frailty Toolset. SPPB: Short Physical Performance Battery. AKI: Acute Kidney Injury.

Beyond direct interventions for frailty and sarcopenia, future perspectives include advancements in pharmacological treatments for patients with AS. The recently published DAPA-TAVI trial [74] stands out in this regard, as it demonstrated the prognostic benefit of dapagliflozin in patients with severe AS. This study is particularly noteworthy given the specific pathology and baseline characteristics, especially the advanced age, of the included patients. This outcome suggests a promising avenue for pharmacological modulation in a patient group that is traditionally managed primarily through mechanical intervention. In this context, further research is anticipated to lead to the development of novel molecules or the re-evaluation of existing medications in new therapeutic scenarios to further improve the prognosis and long-term outcomes in patients with severe AS. Similarly, a randomized clinical trial (RASTAVI) is being conducted to assess whether the administration of angiotensin-converting enzyme inhibitors (ACEIs) improves 3-year survival in hypertensive patients undergoing this intervention [75]. This work will confirm the hypothesis put forward by observational studies, such as the one conducted by Pascual I et al. (2020) [76], Fischer-Rasokat et al. (2022) [77], and the EffecTAVI registry [78], which found a reduction in all-cause and cardiovascular mortality at 2 years when ACEIs or ARBs (angiotensin receptor blockers) were administered after TAVR. At present, in the first year of follow-up, statistical significance has not been achieved for the primary endpoint, although a reduction in the rate of hospitalizations and ventricular volume has been observed. These integrated approaches, which combine interventional refinements with targeted geriatric care and innovative pharmacotherapy, are expected to redefine comprehensive management strategies for patients undergoing TAVR. Figure 2 illustrates insights and a global overview of TAVR in older adults.

Artificial Intelligence (AI) and Machine Learning (ML) are emerging frontiers in the management of transcatheter aortic valve replacement (TAVR). Multiple machine learning algorithms have been developed to predict TAVR outcomes, with a particular focus on cerebrovascular events [79]. These approaches integrate multimodal imaging data to enhance procedural planning and risk assessment [80] and improve predictive accuracy beyond established clinical scores, such as the EuroSCORE II [81,82]. Such technological advancements hold promise for refining clinical decision-making and personalizing patient management. Ongoing investigations aim to validate these AI-driven tools and facilitate their integration into standardized clinical pathways [83].

Key points:

- Research into strategies after TAVR to mitigate the onset of geriatric syndromes has recently increased. Two examples are the PERFORM-TAVI trial, which is evaluating the effect of exercise and nutritional approaches to reverse frailty, and the TELE-FRAIL TAVI study, which is testing the impact of telehealth-based multidisciplinary interventions (nutrition, exercise, education) on functional recovery in elderly patients after TAVR.
- Pharmacological management after TAVR is also a trending topic in research, with ongoing studies such as the DAPA-TAVI trial, which suggests that dapagliflozin may improve prognosis in elderly patients with severe AS, and the RASTAVI trial, which is currently investigating whether ACEIs could reduce adverse outcomes after TAVR.
- Artificial intelligence and machine learning models are emerging tools that enhance risk stratification, procedural planning, and personalized follow-up in TAVR, offering a forward-looking perspective for clinical integration.

Clinical implications:

- Multicomponent interventions after TAVR may improve functional recovery and quality of life in older adults.
- Pharmacological therapy after TAVR can improve procedural outcomes.
- AI-based predictive models can augment clinical decision-making and individualized patient management in TAVR care.

Research Gaps:

- Cost-efficiency of multicomponent interventions after TAVR should be investigated.
- Large-scale randomized clinical trials should be performed to integrate multidisciplinary management of older adults after TAVR, including exercise, nutrition, education, and pharmacological approaches.
- Validation and clinical integration of AI/ML tools require further prospective studies to confirm their predictive accuracy and clinical utility.

## 6. Conclusions

TAVR has revolutionized the management of severe AS, offering a less invasive and highly effective treatment option, which is particularly crucial for the elderly population. Its widespread adoption has significantly improved patient outcomes, extending survival and enhancing quality of life across diverse risk profiles.

The utility of biomarkers and advanced imaging techniques is paramount for optimizing TAVR patient care. Markers such as NT-proBNP and CA125, along with imaging parameters like LVGLS and ECV, should be used to assess cardiac remodeling, predict prognosis, and evaluate therapeutic response. The integration of new serum biomarkers into clinical practice will enable a personalized approach to patient selection for the procedure and post-TAVR management guidance.

Crucially, the increasing applicability of TAVR in an aging demographic has brought the profound impact of geriatric syndromes like frailty, delirium, and sarcopenia to the forefront. These conditions are independent determinants of adverse post-procedural outcomes. In summary, while TAVR improves the quality of life in older patients, an integrated multidisciplinary approach, pre-procedural identification of high-risk individuals, and early detection and management of geriatric syndromes are crucial to optimize outcomes and functionality.

Future directions in TAVR will undoubtedly involve continued refinement of interventional techniques, deeper integration of biomarker-driven strategies, a strong emphasis on comprehensive geriatric assessment, and tailored multicomponent interventions to ensure the best possible long-term benefits for this growing patient population.

Clinical Key points:

- TAVR has been proposed as the standard treatment for AS, especially in elderly patients, owing to its safety and efficacy profile.
- Cardiac remodeling must be assessed using both serum biomarkers and imaging parameters, as they can help optimize procedural planning and management after TAVR.
- Geriatric syndromes such as frailty, delirium, and sarcopenia increase adverse outcomes; therefore, prevention, early detection, and thorough management are imperative.
- Early identification of high-risk patients and multidisciplinary management are crucial for improving procedural outcomes, functional recovery, and long-term results.
- Evidence-based medicine is the cornerstone of obtaining maximum benefits for patients. Ongoing trials will yield tools for optimizing patient outcomes after TAVR.

## Figures and Tables

**Table 1:** Cardiac biomarkers related to cardiac remodeling. Origin, function, and prognostic value in patients undergoing TAVR.

Biomarker	Origin	Function	Prognostic Value
NTpro-BNP	Released by ventricular cardiomyocytes in response to parietal stress	Inhibition of RAS and SNS for natriuresis and arteriolar vasodilation. Reduction of adverse cardiac remodeling and/or increase of reverse cardiac remodeling	Elevated baseline levels: Not clear if predictor of worse prognosis in terms of HF hospitalization or all-cause mortality No reduction of levels after TAVR: Increase in all-cause mortality.
CA125	Glycoprotein mainly released by mesothelial cells of the peritoneum, pericardium, and pleura.	Non-specific tissue irritation marker. Useful in oncological processes and widely used in heart failure decompensation, especially if pleural effusion or ascites	Elevated baseline levels: Increase in all-cause mortality or MACE. No reduction of levels after TAVR: Increase in all-cause mortality or MACE.
SST2	Released by immune cells in inflammatory processes and by cardiomyocytes in response to parietal stress	Inhibits IL-33/ST2 signaling system to prevent myocardial fibrosis and hypertrophic adverse remodeling	Elevated baseline levels: Increase in all-cause mortality. No reduction of levels after TAVR: Increase in all-cause mortality. * Its addition to the STS-score in patients undergoing TAVR could improve prognostic value for all-cause mortality and/or MACE.
GDF-15	Cytokine of the transforming growth factor-B family, released by macrophages, adipocytes, and cardiomyocytes, among others.	Activates MAP-K to regulate TGF-β1/SMAD3 axis, reducing inflammation and oxidative and myocardial stress. In myocardial tissue, it has anti-hypertrophic and antifibrotic effect.	Elevated baseline levels: Lower reversal remodeling after TAVR and an increase in all-cause mortality and MACE. No reduction of levels after TAVR: Increased all-cause mortality and MACE.
Galectin-3	Lectin released by monocytes, macrophages, epithelial cells, and cardiomyocytes, among others.	Regulation of signaling pathways related to cell adhesion, cell proliferation, and inflammation. In the myocardial tissue, related to inflammation, fibrosis, and hypertrophy.	Elevated baseline levels: It might be a predictor of lower reversal remodeling after TAVR. Predictor of increased all-cause mortality and MACE only in the presence of elevated NTproBNP values.

NTproBNP: pro-peptide of brain natriuretic peptide. RAS: renin-angiotensin system. SNS: sympathetic nervous system. BNP: brain natriuretic peptide; TAVR: transcatheter aortic valve replacement. CA 125: cancer antigen 125. MACE: major acute cardiovascular event. IL: interleukin. SST2: soluble isoform of Suppressor of Tumorigenicity 2. STS-score: Society of Thoracic Surgeons score. GDF-15: growth differential factor-15. TGF-B: transforming growth factor B. MAP-K: mitogen-activated protein kinase.

**Table 2:** Illustrates the prognostic value of the cardiac imaging parameters. Cardiac imaging parameters related to cardiac remodelling and prognostic value.

Imaging Feature	Description	Measured	Prognostic Value
LVEDD LVESD LVEDV LVESV	Left ventricular end-diastolic diameter (LVEDD) Left ventricular end-systolic diameter (LVESD) Left ventricular end diastolic volume (LVEDV) Left ventricular end systolic volume (LVESD)	Transthoracic echocardiography: LVEDD and LVESD: long parasternal long axis LVEDV and LVESV: biplane measuring: 2 and 4 chambers/3D echocardiography or Cardiac magnetic resonance	Lowering of the parameters 1 month–1 year after TAVR favors cardiac reverse remodeling, and improvement of HF-related quality of life assessed by KCCQ.
LVMI	Left Ventricular mass index: estimation of the weight of the left ventricle LV mass formula: 0.8 × (1.04 × (((LVEDD + IVS +PW)^3^ − LVEDD^3^))) + 0.6 LV mass index = LV Mass/Body surface area	Transthoracic echocardiography: IVS: interventricular septum diameter (diastolic) PW: diameter of the posterior wall of the left ventricle (diastolic)	
Peak velocity of mitral E wave	Peak velocity of rapid passive left ventricular diastolic filling	Pulsed-wave doppler through the mitral valve, positioned at the level of the junction of the leaflets	
Peak velocity of mitral e’ medial or lateral wave	Peak velocity of the medial or lateral mitral annulus during rapid left ventricular diastolic filling	Tissue doppler waveform placed immediately above the lateral or medial section of the mitral annulus	
E/e’ ratio	Ratio of the peak velocities of the previously described waveforms	Ratio of the peak velocities of the previously described waveforms	
LVGLS	Left Ventricular Global Longitudinal Strain Measurement of left ventricular cardiac muscle deformation from basal to apical using the speckle tracking technique.	Transthoracic echocardiography, using 4-chamber, 2-chamber, and 3-chamber echocardiographic apical views. Cardiac Computed Tomography.	Decreased LVGLS prior to TAVR: Increased all-cause mortality and MACE. No increase in LVGLS after TAVR: Increased all-cause mortality and MACE.
ECV	Extracellular volume of the myocardium. Representation of the myocardial interstitial space may be an indicator of cardiac fibrosis. It has been associated in the field of HF with cardiac events such as mortality or admission for HF.	Cardiac Magnetic Resonance (T1 mapping) Cardiac Computed Tomography	Increased ECV prior to TAVR: Increased all-cause mortality and MACE.
LA strain	Left atrial deformation during the cardiac cycle measured by speckle tracking technology	Transthoracic echocardiography, using a 4-chamber apical view.	No increase in LA strain after TAVR: Increased all-cause mortality and MACE.
RVLS	Right ventricle myocardial deformation during the cardiac cycle measured by speckle tracking technology	Transthoracic echocardiography, using a 4-chamber apical view.	No increase in RVLS strain after TAVR: Increased all-cause mortality and MACE.

LVEDD: Left ventricular end-diastolic diameter. LVESD: Left ventricular end-systolic diameter. LVEDV: Left ventricular end diastolic volume. LVESD: left ventricular end systolic volume. HF: Heart failure. TAVR: Trans-aortic valve replacement. KCCQ: Kansas City Cardiomiopathy Questionnaire. LV: Left ventricle. LVMI: Left ventricle mass index. IVS: Interventricular septum. PW: Posterior Wall of left ventricle. LVGLS: Left ventricular global longitudinal strain. MACE: major acute cardiovascular events. ECV: Extracellular volume. LA: Left auricle. RVLS: right ventricle longitudinal strain.

**Table 3:** Key studies evaluating TAVR outcomes in nonagenarians.

Author, Journal, Year	Population (Comparative Groups)	Endpoints Explored	Main Findings
Abramowitz et al., Am J Cardiol, 2015 [52]	136 patients ≥ 90 years (mean age 92.4) vs. 598 < 90 years (mean age 79.7)	Procedural success, 30-day and 1-year mortality. Major and minor complications	Similar success, mortality, and major complications. Minor vascular complications were more frequent in nonagenarians.
Zadrozny et al., J Cardiol, 2021 [53]	2336 patients (153 ≥ 90 years; 6.6%) vs. younger patients	Stroke, PPI, AKI, anemia, major bleeding, residual aortic insufficiency	No significant differences in periprocedural complications.
Liu et al., J Interv Cardiol, 2019 [54]	Meta-analysis of 5 studies: 25,371 ≥ 90 vs. 21,442 < 90 years	30-day and 1-year mortality. Vascular complications, bleeding, stroke, PPI, AKI	Higher mortality, bleeding, stroke, and vascular events in the ≥90 group; Similar PPI and AKI. TAVR still superior to conservative treatment.
Demir et al., J Invasive Cardiol, 2022 [55]	Meta-analysis of 23 studies (2012–2019), 78,858 total; 16,094 ≥ 90 (20.4%)	Perioperative and 1-year mortality	Perioperative mortality 6.1% (lower than the STS-predicted 10%). One-year mortality 20.5% (better than 40% with conservative care).

## Data Availability

Not applicable. No new data were created or analyzed in this study. Data sharing is not applicable to this article.
